# Testing in the workplace: finding a path through a pandemic

**DOI:** 10.4155/bio-2021-0241

**Published:** 2021-11-18

**Authors:** Jameson Briscoe

**Affiliations:** ^1^BioAgilytix Labs, Durham, NC 27613, USA

**Keywords:** COVID-19, laboratory, workplace testing

The past year marked what for many is the most significant event they have yet lived through. The global scale of the COVID-19 pandemic was mind bending; the gravitas further cemented by its continued duration. Every person’s experience is unique, but several common threads exist, including the spotlight it would shine on the pharmaceutical industry. This is a somewhat reductive statement as there are so many facets to consider within the space, but I think it is appropriate as it was portrayed that way in most media. I was given an opportunity to be a part of this, working at BioAgilytix as a Business Development Assistant selling COVID-19 testing services. I would see not only this side, but as a student at William Jewell College where I participated in ‘Operation Safe Campus.’ This testing/safety regimen included testing by MRI Global very similar to BioAgilytix’s own testing program. I am uniquely positioned to reflect on how the pandemic spotlighted the pharmaceutical industry, and what that looked like.

31 December 2019 marked the beginning of the progression of the SARS-CoV-2 virus with the first official case in Wuhan, China. However, it was not until 9 January that the World Health Organization (WHO) began to suspect there could be a novel coronavirus causing the mysterious respiratory symptoms. The WHO began development of a detection assay for the virus; however, in many countries, where the capabilities to develop their own tests existed, the WHO-developed tests were not adopted [1]. The reasoning behind this decision remains unanswered in many countries, such as the USA. Later experience demonstrated that testing is one of the most effective methods for mitigating the spread of SARS-CoV-2, yet, in October 2021, over 700,000 lives have been tragically lost in the USA alone [2]. The USA has lagged in COVID-19 mitigation efforts, with similarly developed nations reporting 40% fewer associated deaths per capita. How this massive failure occurred is, and will remain, a pertinent point as epidemiologists and crisis aversion experts fight to prevent future pandemics. Certainly, an earlier commitment to and roll-out of more effective testing procedures would have minimized the impact.

While the pandemic period has given rise to some of the fastest scientific discoveries and developments, such as development of the new mRNA vaccines, it was also characterized by overly politicized and slow, ‘too little too late’ administrative decisions. One example of this within the USA was the seemingly slow decision to allow Clinical Laboratory Improvement Amendments (CLIA)-approved labs (which perform testing in human specimens) to design their own testing kits [3]. There seemed to be a lack of coherent messaging on the responsibilities of both public and private institutions regarding testing, quarantine and other pandemic response measures. The necessity of the development of independently developed CLIA-certified lab kits became even more critical when the US CDC realized their own assay was producing unreliable test results [4]. There was eventually the reduction in regulatory requirements through aggressive application of the US FDA’s Emergency Use Authorization process for tests; however, testing remained bottlenecked for most people throughout 2020.

In lieu of a national lockdown, the CDC created a set of guidelines to ensure that people could continue their lives with some semblance of normalcy. The guidelines developed were often unclear and had a number of conflicting points. One example of this was stated in the official testing strategy that “Workers in critical infrastructure sectors may be permitted to work if asymptomatic after potential exposure to a confirmed case of COVID-19, provided that worker infection prevention recommendations and controls are implemented.” [5]. The combination of ambiguity and weak enforcement measures for these guidelines meant that adherence to the guideline was inconsistent at best. Individuals who were in-person for a job already exacerbated the risk of infection and the lack of enforceable guidelines made it much worse [6]. Many businesses opted for daily health screens, checking in with workers to see if they had displayed any symptoms. As it has been well known since early in the pandemic that people can be both asymptomatic and contagious, this approach was destined to be only marginally successful [7]. Testing, therefore, is the only way to confirm whether a workplace is safe. The failure of the guidelines based on assessing symptoms is exemplified by early outbreaks in high-density workplaces like meat-packing plants. It has been reported that counties with beef and pork meat-packing plants had infection rates twice that of the surrounding counties [8].

The USA was slow to adopt widespread testing protocols when compared with other developed nations. Reports from May 2020 showed one of the lowest reported testing rates [1]. Google’s COVID-19 tracking, supported by data from The New York Times, showed that June 2020 would begin the second dramatic rise in infections. Companies unable to provide physical distancing, work from home or other safety measures and without a proper plan in place for testing in a workplace would see limited success with preventing the spread of COVID-19 [7]. Several independent labs who did not normally work in this testing space sought to remedy this; as they already possessed the necessary equipment and expertise to perform testing. Many also felt a moral obligation to support testing efforts as an additional motivation.

I was lucky enough to have an opportunity to participate in this as both a consumer and distributor. As a student, my college was working with an independent lab for a number of reasons, most important of which was the reliability of signing with a specific testing partner, and so therefore, having the ability to test at will with guaranteed turnaround times. This meant there was greater ability to contact trace, limited exposure once showing symptoms and a greater ability to quarantine those who were asymptomatic. Having a plan as William Jewell did seemed to be the exception. When I began to try to sell to colleges, they either had plans or, more commonly, they were content to let students make their own decisions regarding pandemic safety.

At the same time, I was working at BioAgilytix, a global bioanalytical contract research organization, as a Business Development Assistant for their COVIDence™ program. The plan was to fill in the gaps left by large diagnostic labs like LabCorp and Quest whose testing capacity was overwhelmed. We were able to work more closely with clients on not only providing testing but also managing any outbreaks that would occur within their workplaces. The benefits of workplace testing were easily seen, with studies reporting daily PCR testing reducing infection rates by around 60%, biweekly by around 40% and weekly testing by around 20% [9]. On top of the benefit to employee safety, simulated models showed that universal testing was also a cost-effective strategy for business because there were less infections, hospitalizations and deaths.

Despite the proven benefits of testing, only businesses in certain industries were willing to discuss a testing plan. Although BioAgilytix is a direct business-to-business company, it has always worked in the biotech industry so a new strategy to connect with businesses was required. The outreach to companies involved in hospitality, the food industry, colleges and more would require a different approach. Traditionally, contract research organizations work to support clients who have specific drug development testing needs, but during the pandemic, we were faced with explaining the need for testing to prospective clients, many of whom – surprisingly – did not want to discuss anything even remotely related to COVID-19. This would all become more evident as time went on, with my team realizing our originally planned direct outreach approach was ineffective. It proved exceedingly difficult to convey the credibility of our CLIA-accredited laboratory they had not heard of because we sit outside of their industry and do not typically work direct-to-consumers.

There are a number of possible reasons for why these conversations were so difficult, but perhaps the most significant was the misinformation that had surrounded anything related to SARS-COV2 leading to mistrust and misunderstanding. The media was so oversaturated with pandemic news that people no longer trusted what they did not understand. Outreach to help educate and assist in constructing a testing plan to help support their business was simply not a conversation that was of interest in most cases. For instance, a casino owner in Las Vegas who had everything to gain from implementing a comprehensive testing plan for his employees unsurprisingly had no idea who BioAgilytix was and with no credibility attached to that name that would convince them to spend time talking to us, a conversation simply did not materialize. Crisis fatigue was widespread in the late summer and fall of 2020, and it would be unfair to expect anything otherwise [10].

Personally, I was able to see how successful implementing a plan like this could be. My *alma mater* had set up their own plan, which included regular surveillance testing, contact tracing and quarantine protocols. Their proactive approach allowed students to have a much safer environment, especially when looking at it relative to many institutions that would struggle with localized and sometimes even widespread outbreaks. William Jewell both communicated and followed a well defined plan, giving students faith in the institution to keep them safe and a willingness to abide by the system. Their transparency allowed us to see the success of following the plan, as the surveillance testing consistently showed positive testing percentages significantly lower than the surrounding community.

However, programs like COVIDence and others did find success in certain segments. Industries that implemented very significant programs for testing included entertainment, professional sports and education. It was not uncommon for the entertainment and professional sports (such as the NBA’s ‘bubble’) industries to conduct weekly, or in some cases daily, testing to continue their productions in a healthy way. In higher education, we saw many colleges and universities conducting testing when restarting school or when outbreaks would occur. These programs met with mixed success, but as imperfect as some implementations might have been, what should be noted is that they did take advantage of the testing resources.

Like so many aspects over the course of the pandemic, change came fast. In the testing industry, we saw it evolve slowly at first as tests were developed, policies implemented and a whole new COVID-19 testing industry was born. This new testing industry and approach evolved quickly as well. Laboratories filled a short-term need while capacity at the global central labs and hospitals was expanded over the course of 2020. Testing collection procedures and testing sites also evolved to enable simple drive-thru or walk-in collection approaches with logistics networks to get samples quickly and efficiently to laboratory testing centers. The need for the diversity of labs involved in testing waned and enabled them to refocus on their more traditional emphasis; in the case of BioAgilytix, resources were refocused to the support of clinical trials in drug development including several COVID-19 treatments.

COVID-19 has been a tremendous challenge for society in many ways. The danger of misinformation, or a lack of information at all, was shown in the previous year and a half. Governments and individual businesses struggled and continue to struggle to find their answers to how to deal with it. Those that were in the testing business looked for ways to help reduce the spread through their services. Other businesses looked at how to keep their people safe and continue to operate to keep their people employed. What will be important is that we realize our mistakes. The world cannot afford to mismanage disasters to this magnitude. You should not need an advanced degree or a position in the pharmaceutical industry to understand a pandemic response. More people need coherent messaging so that the next global pandemic, whether it is in 5 years or 50, we will be better prepared.
